# Comparative Analysis of Trifluoracetic Acid Pretreatment for Lignocellulosic Materials

**DOI:** 10.3390/ma16155502

**Published:** 2023-08-07

**Authors:** Sara Piedrahita-Rodríguez, Stéphanie Baumberger, Laurent Cézard, Jhonny Alejandro Poveda-Giraldo, Andrés Felipe Alzate-Ramírez, Carlos Ariel Cardona Alzate

**Affiliations:** 1 Institute of Biotechnology and Agribusiness, Chemical Engineering Department, National University of Colombia, Manizales 170003, Colombia; spiedrahitar@unal.edu.co (S.P.-R.); japovedag@unal.edu.co (J.A.P.-G.);; 2 Institut Jean-Pierre Bourgin (IJPB), INRAE, AgroParisTech, University Paris-Saclay, 78000 Versailles, France; stephanie.baumberger@inrae.fr (S.B.); laurent.cezard@inrae.fr (L.C.)

**Keywords:** C5–C6 sugars, CapEx and OpEx, lignocellulosic pretreatment, TFA pretreatment, techno-economic assessment

## Abstract

Lignocellulosic materials are usually processed toward C5 and C6 corresponding sugars. Trifluoroacetic acid (TFA) is a pretreatment method to solubilize hemicellulose to sugars such xylose without degrading cellulose. However, this pretreatment has not been compared to other processes. Thus, this paper focuses on the techno-economic comparison of the C5–C6 production of C5–C6 as raw materials platforms using non-centrifuged sugarcane bagasse (NCSB) and *Pinus patula* wood chips (PP). Hydrolysates using TFA 2.5 M as an acid were characterized through HPLC regarding arabinose, galactose glucose, xylose, and mannose sugars. Then, simulations of the processes according to the experimental results were done. The economic assessment was performed, and compared with some common pretreatments. The mass and energy balances of the simulations indicate that the process can be compared with other pretreatments. From the economic perspective, the main operating expenditures (OpEx) are related to raw materials and capital depreciation due to the cost of TFA corrosion issues. The processes showed a CapEx and OpEx of 0.99 MUSD and 6.59 M-USD/year for NCSB, and 0.97 MUSD and 4.37 MUSD/year for PP, considering a small-scale base (1 ton/h). TFA pretreatment is innovative and promising from a techno-economic perspective.

## 1. Introduction

Biomass transformation has been profiled as a competitive and promising alternative for fossil fuel replacement for obtaining chemical products and energy carriers. However, some issues regarding process modeling must be overcome, such as bioprocess design, continuous process systems, processing scale analysis, economic limitations, and environmental regulations [1]. The challenge is to propose efficient fractionation schemes for lignocellulosic biomass solubilization. The C5 and C6 sugar platforms obtained from biomass are important in the biotechnology industry after feedstock pretreatment, as these sugars can be considered as building blocks in several processes and promising molecules for developing a sustainable industry [2,3]. The objective of pretreatment is to disrupt the complex structure of the biomass, to improve the further processing required by using each platform [4]. In the case of cellulose, pretreatments aim to enable the accessibility of this platform for further transformation (e.g., through enzymatic saccharification). On the other hand, pretreatment aims to degrade the hemicellulose fraction to obtain sugars (mainly C5). Finally, pretreatment influences the solubility of lignin; thus, making it easier to use it (towards interesting compounds or as an energy source) [5].

Lignocellulosic materials have considerable amounts of hemicellulose, cellulose, and lignin. Especially, the heterogeneous hemicellulose polymers are striking for their readiness to be degraded. Examples of lignocellulosic materials are pine wood chips and non-centrifuged sugarcane bagasse. These residues are found in abundance in countries such as Colombia. Characterizations of 23–34% of hemicellulose have been reported for these raw materials, differing mainly in lignin content [4]. Pine, a woody biomass, tends to have a more rigid structure than non-centrifuged sugarcane bagasse due to the lignin content (between 25–40%, compared to 10–17% for bagasse) [5]. This difference is because hemicellulose is more available for the case of bagasse than pine. Cellulose content is also important due to its wide applications. In fact, cellulose is used as a platform for fermentation processes or as nanocomposite for different uses [6]. Pretreatments can be classified as chemical, biological, thermal, thermochemical, physical, and physicochemical. Dilute acid (mainly with H_2_SO_4_), alkaline, hot water, ultrasound, enzymatic, and steam explosion are the most common schemes for lignocellulosic feedstocks [7,8]. However, pretreatment is one of the major bottlenecks in biotechnological processes due to some technical restrictions regarding the energy requirements, operating conditions, waste treatment, inhibitory compound formation, and product degradation [9].

Trifluoroacetic acid (TFA) is an organic acid (pKa = 0.5) used in industry as a raw material in organic synthesis [9]. In addition, properties such as its boiling point (72.4 °C) allow the recovery of TFA with simple operations (e.g., evaporation). Therefore, TFA has been proposed as an alternative for biomass pretreatment to obtain sugars from the hemicellulose fraction [9]. Using TFA achieves high yields in obtaining soluble sugars and a lower degradation from cellulose compared to other acids and bases [10]. TFA has been evaluated and shown not to affect cellulose under normal conditions (unlike most mineral acids). Moreover, an advantage of the TFA is related to easy recovery of this organic acid by evaporation avoiding detoxifications stages (which originate from the formation of salts or streams that require special treatment for their disposal) [11]. TFA has been used, for example, as an effective dispersing medium for cellulose nanocrystals. These applications have shown the solvent properties and suspension stabilizing potential of TFA, among other properties [12].

Pretreatments of lignocellulosic material have been deeply studied and several processes have been standardized towards kinetic optimization [13,14]. However, the deep analysis of pretreatment alternatives is crucial due to the complexity of biomass processing and the requirements of the most technically, energetically and economically convenient in terms of prefeasibility. Thus, TFA as an acid pretreatment is worth analyzing because it is necessary to know its behavior with different raw materials. Moreover, to check the operating conditions that have been reported for other acids is necessary to see if they are viable for TFA, and determining whether they are adequate or not [15]. It would also be interesting to optimize the process and complement it with analyses of economic, environmental and even social spheres. Although TFA has been used in industry, there have been no rigorous studies focused on pretreatment. This paper proposes the experimental and simulation study of TFA as acid pretreatment of two lignocellulosic feedstocks and compare the process performance with other well-established technologies, such as dilute sulfuric acid, dilute HCl, and steam explosion pretreatments, based on data from the literature. In addition, this paper develops the techno-economic analysis of TFA pretreatment considering the capital expenditures (CapEx) and operating expenditures (OpEx) of the scheme.

## 2. Materials and Methods

### 2.1. Raw Materials and Reagents

The non-centrifuged sugarcane bagasse (NCSB) was obtained from a panela (unrefined whole cane sugar) mill in Samaná, Colombia (5°24′47″ N 74°59′34″ W). Pinus patula wood chips (PP) were obtained from a sawmill company located in Manizales, Colombia (5°03′58″ N 75°29′05″ O). Both residues were collected, dried, and cut into 5–10 cm long pieces. The samples were stored in airtight bags protected from direct light, heat sources, and humidity. Then, the residues were milled to 4 mm for characterization. TFA reagent was used and diluted to 2.5 M concentration.

### 2.2. Chemical Characterization

The raw materials were characterized in terms of chemical composition, such as moisture content (ASTM E871-82), ashes (NREL/TP-510-42622), water and ethanol extractives (NREL/TP-510-42619), cellulose, hemicellulose (Han, 1996), and lignin (NREL/TP-510–42618). The characterization was performed in triplicate following the international standards. The thermal stability of the raw material is important to understand the behavior in the hydrolysis assay. Thus, thermogravimetric analysis (TGA) and derivative thermogravimetric analysis were performed and discussed in this section. Finally, as an important part of the characterization of the raw materials, a proximate analysis was carried out (ASTM E872-82 and ASTM D3172-13). This information allowed us to obtain elemental analysis through correlations based on proximate analysis, according to Shen et al., 2010 [16]. The elemental composition allowed us to determine the empirical formula of both raw material and the molecular weight.

### 2.3. TFA Pretreatment Methodology

Pretreatment of NCSB and PP was performed in triplicate, following the protocol reported by Sipponen et al. (2013) [17]. Previously, an oven was conditioned at 120 °C. Initially, 10 mg of extractive-free and dried raw material was transferred to capped test tubes. Then, 500 µL of 2.5 M TFA was added to each test tube and heated at 120 °C for 2 h. After this time, the samples were airconditioned to room temperature and transferred to centrifugation vessels. Centrifugation was performed for 10 min at 3700 rpm. Subsequently, the supernatants were collected for further analysis in high-performance liquid chromatography (HPLC).

### 2.4. HPLC Analysis

The chromatographic separation was performed in an HPLC (Dionex–Thermo Fisher Scientific^®^) equipped with a CarboPac PA1 m 4.5 × 250 mm column and a CarboPac PA1 4 × 50 mm Guard precolumn, as well as an autosampler AS50 and a gradient pump GP 50. The sample analysis was performed in triplicate. The oven and injector temperatures were adjusted to 20 °C. All of the samples, including standards and the pretreated samples, were treated before HPLC analysis. The supernatants were diluted 500-fold:20 µL of supernatant was transferred to a vial, and 980 µL of water was added. After dilutions, the remaining supernatants were stored at –20 °C. Finally, the vials with the samples were filtered and transferred to the appropriate vials for analysis. The phases for chromatography analysis is 50 mM and 300 mM of NaOH solutions, and the flow was fixed in 1 mL/min. The sugar quantification included fructose, arabinose, galactose, glucose, xylose, and mannose. Appendix A shows the calibration curves.

### 2.5. Process Evaluation

#### 2.5.1. Process Description

**Figure 1:**
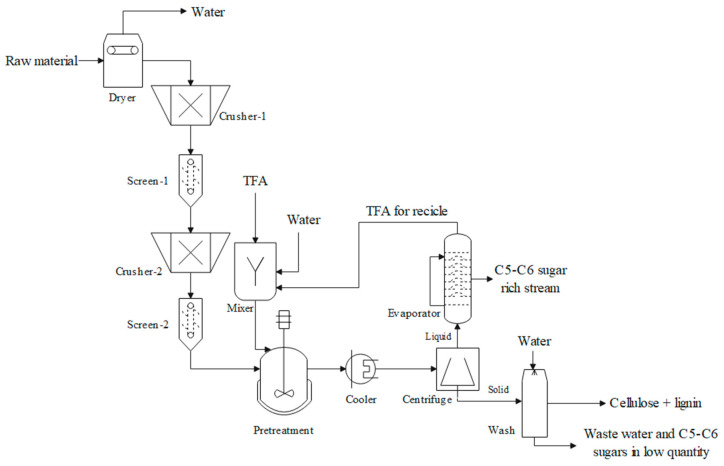
Process flow diagram for the TFA pretreatment of PP and NSCB.

The process flow diagram of the TFA acid pretreatment is shown in Figure 1. The diagram is similar for PP and NCSB, only differing in the drying stage. This process consists of a conditioning stage of the raw material through drying and particle size reduction (mills and sieves) to 1–2 cm, approximately. In the case of PP, the drying stage was carried out by solar radiation, and no equipment is considered in the simulation. However, in the case of NCSB, a convective dryer was necessary before milling. Subsequently, both feedstocks were taken to a reactor for pretreatment. The pretreatment involved 2.5 M TFA at a solid:liquid ratio of 20:1 (*w*/*v*). The production of inhibitory compounds (furfural and 5-hydroxymethyl furfural) was considered from yields reported in the literature [11]. The TFA pretreatment was carried out at 120 °C for 2 h. The experimental conditions were used in the simulation procedure (fixed before a literature review). Subsequently, the outlet stream was centrifuged, and the solid fraction was taken to a washing tank to obtain cellulose and lignin rich stream, which can be used for further processing. On the other hand, the liquid stream was carried to an evaporator to remove TFA and recycle this compound to the process (90%). In this sense, the C5–C6 sugars rich stream will serve as a fermentable sugars stream for further processing.

The process was simulated in Aspen Plus V.9.0 (Aspen Technologies, Inc., Huston, TX, USA) software and the equipment models and conditions used are shown in Table 1. A base of 1 ton/h was used as the raw material flow for the process simulation. The experimental data were used as input for the simulations. The Non-Random Two Liquids and Hayden-O’Connell (NRTL-HOC) thermodynamic method was applied in this case due to the conditions of the process (pressures and temperatures) and the presence of organic acids in vapor phase. The mass and energy balances were obtained and served to analyze the processes at the technical, energetic, economic, and environmental levels.

#### 2.5.2. Techno-Energetic Assessment

The mass and energy balances obtained from the simulation in Aspen Plus V.9.0 (Aspen Technologies, Inc., USA) were used to determine the technical analysis of the processes through the calculation of mass and energy indicators [18]. The indicators are presented in Equations (1)–(5), where ${\dot{m}}_{Product,i}$ is the mass flow of the products, ${\dot{m}}_{Rawmaterial}$ is the mass flow of the raw material, ${\dot{m}}_{in}$ is the mass flow of the in-streams, and Q and W are the total energy consumption of the process.

**Mass indicators:**

(1) $$\mathrm{Product}\;\mathrm{yield}\;(Y_{P}):\;\;\;\;\;\;\;\;\;\;\;\;\;\;\;\;\;\;\;\;\;\;Y_{P}=\frac{\sum {\dot{m}}_{Product,i}}{{\dot{m}}_{Raw\;material}}$$

(2) $$\mathrm{Mass}\;\mathrm{loss}\;\mathrm{intensity}\;(PMI):\;\;\;\;\;\;\;\;\;\;\;\;\;\;\;PMI=\frac{\sum {\dot{m}}_{i}^{in}}{\sum {\dot{m}}_{Product,i}}$$

(3) $$\mathrm{Mass}\;\mathrm{loss}\;\mathrm{index}\;(MLI):\;\;\;\;\;\;\;\;\;\;\;\;\;\;\;\;\;\;\;\;\;MLI=\frac{\sum {\dot{m}}_{i}^{in}-{\dot{m}}_{Product}}{\sum {\dot{m}}_{Product,i}}$$

(4) $$\mathrm{Renewable}\;\mathrm{material}\;\mathrm{index}\;(RMI):\;\;\;\;\;\;\;\;\;\;\;RMI=\frac{\sum_{i\_1}^{N}{\left({\dot{m}}_{i}^{in}\right)}_{renewable}}{\sum_{i=1}^{N}{\dot{m}}_{i}^{in}}$$

**Energy indicator:**

(5) $$\mathrm{Specific}\;\mathrm{energy}\;\mathrm{consumption}\;(S_{EC}):\;\;\;\;S_{EC}=\frac{\dot{Q}+\dot{W}}{{\dot{m}}_{Raw\;material}}$$

#### 2.5.3. Economic Assessment

The economic assessment was performed using an Aspen Process Economic Analyzer V.9.0 (Aspen Technologies, Inc., USA). The mass and energy balances obtained from the simulation were used as input data. The process was considered for 24 h continuously. The capital expenditures (CapEx) of the processes were determined through equipment sizing based on flow capacities. Additionally, the detailed economic design methodology described by Rueda et al. (2022) [19] was applied, since this methodology improves the CapEx estimation, considering mechanical costs, instrumentation, civil works, piping, electrical costs, firefighting costs, and contingency costs. Moreover, the operating expenditures (OpEx) were calculated considering Colombian economic conditions as the basis for the analysis and a straight-line method of depreciation calculation. Table 2 shows all the values considered, including raw materials and inputs costs. The analysis considered 350 days/year of operating time because the scale context is small.

CEPCI: Chemical Engineering Plant Cost Index

The CapEx for these types of processes included equipment, installation, instrumentation and control, and electrical costs. However, a process scale analysis was performed to evaluate the contribution of the costs in the CapEx for a large-scale process. Equation (6) shows the division of cost in the CapEx calculation, and all of the share percentages can be seen in Rueda-Duran et al. (2022) [19], where Equipment Cost (EC) was determined with an Aspen Plus Economic Analyzer; Total Direct Cost (TDC) considers the costs of installation, instrumentation and control, piping, electrical buildings including services, yard improvements, and service facilities; Total Indirect Cost (TIC) considers engineering and supervision costs, construction expenses, and legal expenses; and finally, function of total direct and indirect cost (FTDIC) includes the contractor’s fee and contingency.

(6) $$\mathrm{CapEx}=\mathrm{Total}\;\mathrm{Equipment}\;\mathrm{Cost}\;\left(\mathrm{TEC}\right)=\sum \mathrm{Equipment}\;\mathrm{Cost}\left(\mathrm{EC}\right)+\sum \mathrm{Total}\;\mathrm{Direct}\;\mathrm{Cost}\left(\mathrm{TDC}\right)\;+\sum \mathrm{Total}\;\mathrm{Indirect}\;\mathrm{Cost}\;(\mathrm{TIC})+\sum \mathrm{As}\;\mathrm{function}\;\mathrm{of}\;\mathrm{total}\;\mathrm{direct}\;\mathrm{and}\;\mathrm{indirect}\;\mathrm{cost}\left(\mathrm{FTDIC}\right)$$

## 3. Results

### 3.1. Experimental Results

#### 3.1.1. Characterization

**Figure 2:**
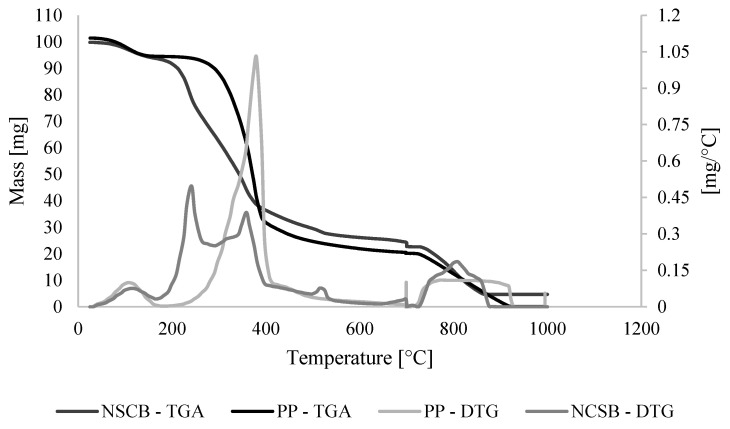
TGA and DTG diagrams for PP and NCSB.

The moisture, ash, and extractives content in NCSB and PP were 7.26%, 1.01%, 20.40%, and 11.19%, 0.41%, 11.55%, respectively. Cellulose, hemicellulose, and lignin content in NCSB were 33.27%, 19.90%, and 18.16%, and 33.00%, 17.56%, and 26.29% in PP. More than 50% of both raw material’s compositions correspond to the lignocellulosic matrix. In this paper, the lignocellulosic composition was comparable with the literature. However, small differences were found in the sugarcane bagasse reported by Jin et al. (2020) [25] and Wang et al. (2022) [26]. Ponce et al. (2021) [27] also report some compositions of sugarcane bagasse of 58.76, 17.67, 12.74, and 1.04% for cellulose, hemicellulose, lignin, and moisture. The variation in the composition is related to the type of crop, climatic conditions, and use of agrochemicals. On the other hand, García-Velásquez and Cardona Alzate (2019) [24] reported a chemical composition of 9.21, 44.78, 23.75, 20.22, 11.0, and 0.25% of moisture, cellulose, hemicellulose, lignin, extractives, and ash for PP. Although the origin of the reported PP is the same as that shown in this study, differences in composition are due to variations in sampling time or even changes in wood processing [24]. Despite this, the distribution of components within the PP composition remains the same, with a higher cellulose content. Regarding both raw materials, there are significant differences in their characterization. PP, a wood raw material, presents a more rigid structure, explained by the higher lignin content than NCSB. In this sense, it is expected that in the case of NCSB, hemicellulose is much more available for pretreatment. However, conditions such as thermal stability and ultimate composition may influence the behavior in subsequent hydrolysis. The TGA and DTGA analysis of raw materials allows us to understand the composition and behavior (see Figure 2). For NCSB case, two peaks were found before 400 °C. The first one corresponds to the degradation of hemicellulose. For the case of PP, just one peak was established. The first peaks were found at 240 and 380 °C for NCSB and PP, respectively. The results of this peak indicate that the hemicellulose fraction is more available for NCSB than for PP, which would lead us to expect a good performance in processing, such as pretreatment with TFA. The second peak found in NCSB materials corresponds to the degradation of the cellulose fraction, which occurs at approximately 358 °C. As the decomposition temperature range in both cases is wide, it can be deduced that the most representative fractions in raw materials correspond to cellulose and hemicellulose. This is consistent with the results obtained for the chemical characterization. Comparing these results with some reports, it is possible to find that Muñoz et al., 2015 carried out the TGA analysis for pine [28]. The authors performed TGA/DTG analysis at 5, 20, and 50 °C/min heating rates in an inert atmosphere. Pyrolysis was reported to start at 250 °C and end at 370, 400, and 420 °C, respectively, for each rate. This temperature range for decomposition (250–420 °C) suggests that this type of pine contains higher amounts of hemicellulose and cellulose than other species. After 400 °C, slower decomposition was observed due to lignin, which decomposes between 277 and 527 °C [29]. On the other hand, Moretti et al., 2016 performed a thermogravimetric analysis of sugarcane bagasse, reporting high stability because the degradation temperature was 350 °C [30]. Two peaks were reported: the first corresponds to the decomposition of hemicellulose and lignin at approximately 360 °C, and the second corresponds to the decomposition of cellulose at 405 °C. The results indicate the high availability of hemicellulose for this feedstock for the pretreatment stage.

The proximate analysis for the raw materials was performed. The results can be seen in Table 3. In addition, the elemental analysis determined according to the correlations reported by Shen et al., 2010 [16] can be seen. With this information, the molecular weight of PP is 149.28 and 148.56 g/mol for PP and NCSB, respectively. The results serve as input for verifying the simulation calculation of the molecules’ properties.

#### 3.1.2. TFA Pretreatment and Sugars’ Quantification

The TFA pretreatment converted approximately 80% to sugars from the hemicellulose platform in both feedstocks. Regarding pretreatment performance towards C5 sugar production, non-wood biomass is better than woody biomass due to its solubilization potential. NCBS has a cellulose and hemicellulose content similar to PP, but the lignin content is lower, causing the hemicellulose fraction to be much more available for TFA pretreatment. Therefore, more sugar release is expected. At the experimental level, TFA was not recovered due to the quantities used for the test. Even so, it is recommended to consider the recovery of this acid in the simulation to improve the process in technical–economic terms. Bay et al. (2020) [31] reported a final composition of cellulose and hemicellulose of *Pinus radiata* (PR) after pretreatment (48% and 7%, respectively), demonstrating that the conversion of the hemicellulosic fraction is high (more than 60%) and the cellulose fraction remains unvariable. Marzialetti T et al., 2008 [11] reported a degradation of 70% of the hemicellulose fraction during TFA pretreatment of Loblolly pine at 150 °C and pH 1.65, demonstrating a better performance in comparison with mineral acids.

The sugar concentrations founded in hydrolysate are shown in Table 4. The concentration of xylose and glucose were the most representative for both raw materials. The presence of xylose after TFA pretreatment is attributed to the breakage of β-1,4-xyloses bonds linked with arabinose. On the other hand, glucose can be attributed to the hydrolysis of non-crystalline cellulose, β-1,3- and β-1,4- main glucans, or to solubilized sugars from other molecules that may be present in a small proportion (e.g., starch in NCSB or some flavonoids) [32]. This indicates a partial solubilization of the cellulose fraction for both raw materials. Some other authors report sugar content after pretreatments using H_2_SO_4_ or HCl as acid and the results are shown in Table 4.

Berrocal et al. (2004) [33] performed a TFA pretreatment of *Pinus radiata* (PR). The pretreatment conditions vary considerably with this paper, due to the hydrolysis time (12 h vs. 2 h) and temperature (20 °C vs. 120 °C). Initially, the PR evaluated by the authors had a composition of 31.05, 25.99, and 38.96% *w*/*w* of cellulose, hemicellulose, and lignin. Some authors have reported sugar content after pretreatments, such as steam explosion, dilute sulfuric acid pretreatment, and alkaline pretreatment. For example, Chacha et al. (2011) [38] reported PP pretreated by steam explosion yields of 0.02 and 0.04 Ara(-Xylan) and Gal(-Glucomannan), respectively. The reported compositions determine the fraction of cellulose or hemicellulose degraded by the pretreatment. Both yields (defined as C5 and C6 sugar platforms) are low compared to the results obtained in this paper. Lavarack et al. (2002) [39] reported yields below 2% in sugars such as arabinose, glucose, and xylose from pretreatment of sugarcane bagasse (SCB) using several pretreatment conditions with dilute sulfuric acid. Gomez et al. (2014) [32] performed the evaluation of alkaline (with NaOH) and diluted acid (with H_2_SO_4_) pretreatment and showed that, for the case of several raw materials, including SCB, alkaline pretreatment releases mostly C5 sugars, while dilute acid pretreatment reflects higher amounts of C6 sugars at low temperatures. As temperature increases in both pretreatment types, the relative abundance of glucose decreases due to an increase in xylose. It can be evidenced by comparing these results with those obtained with TFA pretreatment at 120 °C, since the concentrations of these sugars are high. Other pretreatments have been reported for PP and SCB. For example, Banerje et al. (2014) [40] performed a hot water pretreatment to SCB, reporting 0.25, 0.03, 0.10, 0.43, and 0.04 g of arabinose, galactose, glucose, xylose, and mannose per g of SCB, respectively. Xu et al. (2006) [41] reported that pretreatment of SCB with HCl improved xylose and arabinose (0.45 and 0.38 g/g SCB) yields. Reyes et al. (2013) [42] obtained 1.8, 14.1, 10.3, 5.0, and 26.7 g of arabinose, galactose, glucose, xylose, and mannose per g of hemicellulose present in PR, using hot water pretreatment.

Nevertheless, one way to compare the TFA pretreatment with the other pretreatments is by determining the severity factor (SF). SF can be determined from pretreatment parameters such as temperature, time, and pH. For this case, the normal SF was implemented, in which only temperature and time are considered as comparative parameters of the pretreatments (see Equation (7)) [43].

(7) $$R_{0}=t*exp\left(\frac{T\left(t\right)-100}{14.75}\right)$$

Table 5 shows the SF of different pretreatments. The SF values show the severity of pretreatments implemented for lignocellulosic biomass. These schemes impact technical and economic aspects such as equipment energy consumption, sizing, and cost. As mentioned in the methodology part, the conditions of the TFA pretreatment were considered according to the literature review. The aim is to compare the SF value with other conditions in pretreatments of lignocellulosic materials. Therefore, high SF values tend to be represented by high temperatures, middle times, and degradation rates. Therefore, the sizing will generate high cost in equipment and high energy demand. However, the variability of pretreatment conditions makes them different. Therefore, what can be analyzed is precisely the effect of these conditions at the technical and economic level (due to the characteristics of the equipment), and not a comparison of them.

### 3.2. Simulation Results

#### 3.2.1. Technical Assessment

The results of the mass and energy indicators for both cases are shown in Table 6. The yields of each product are higher than 20%. The report of the sugar-rich stream considers the yields obtained experimentally for C5 and C6 hydrolyzates sugars. Hence, the yield data correspond to the total sugars (C5 and C6). For both cases, the mass and energy indicators are similar because the initial composition of the raw materials and their yields were also similar. Nevertheless, the fact that the lignin content is higher in PP than in NCSB makes the cellulose and hemicellulose (sugar precursors) more available to be transformed by the action of TFA for the NCSB case. The pretreatment stage generates large amounts of waste, mainly due to water use in washing and diluting the TFA; a similar situation. The waste outlet streams from the evaporator and scrubber are the main contributors to the significantly high MLI (see Table 6). Thus, one of the main bottlenecks can be identified from this perspective. The need to couple a detoxification stage allows a reduction in the waste load. Nevertheless, by-products with no significant commercial value are generated and are an issue for disposal [47]. In the case of TFA pretreatment, it can be recovered without requiring this additional stage, reducing waste stream flow or include a wastewater treatment stage. Also, such streams could be integrated to be treated as a utility within the same scheme. PMI and RMI values are similar for both raw materials due to the conditions assumed for the simulation, according to the experimental results. Since the process does not have self-generation or energy-vector products, the efficiencies are very low and the $S_{EC}$ is high. From the energy perspective, this independent processing section is very demanding, which can be improved by including energy integration. Baral et al. (2017) [9] have reported that the pretreatment stage (biological, acid, and steam explosion, among others) is the most energy-demanding compared to feedstock conditioning, detoxification, or enzymatic saccharification processes. This is due to the temperatures of these processes: usually between 60–200 °C. Additionally, the utilities involved in this process also affect the process, considering the equipment requirements. When high residence times are handled, the energy demand increases compared to continuous and short residence time processes. However, the use of cooling water as a utility, for example, is much less demanding in terms of energy costs than other refrigerants. As a proposal to improve pretreatment energy, the use of the obtained platform products (further transformation) or energy integration through waste streams should be considered.

#### 3.2.2. Economic Evaluation

**Figure 3:**
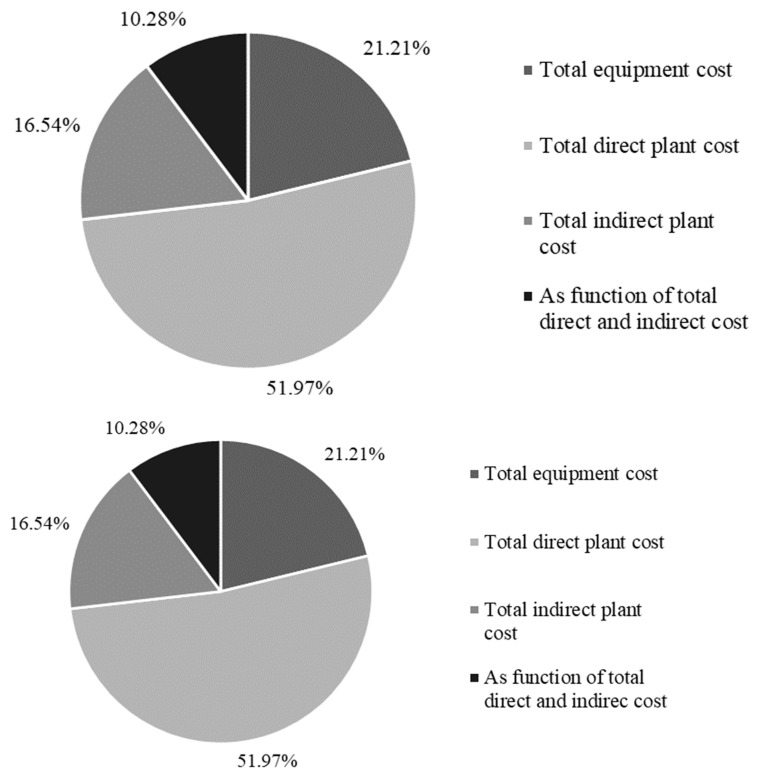
Distribution of CapEx cost in large-scale case of TFA pretreatment of PP and NCSB.

The CapEx and OpEx of TFA pretreatment for NCSB were 0.99 MUSD and 6.59 MUSD/year, and for PP, it was 0.97 MUSD and 4.37 MUSD/year for a small-scale process. Figure 3 shows the distribution of costs in the CapEx calculation for a large scale. For both cases, the TDC represents the highest contribution (over 50%), while the lowest is the FTDIC. The pretreatment process is generally assessed economically in a complete biomass valorization processes (for example, to obtain ethanol). The results obtained in this paper could be compared with reports in which economic viability exists in the entire transformation process (starting from the platform molecules that are the C5 and C6 sugars). For example, Zhao et al. (2015) [48] demonstrated the economic feasibility of obtaining ethanol from corn stover using dilute sulfuric acid as pretreatment. The CapEx is calculated based on direct and indirect costs, and the OpEx, unlike this study, includes waste disposal and ash disposal prices. In this case, the cost of raw materials is lower, so the OpEx is more favorable for the dilute acid pretreatment.

To compare the economic results, to determine the specific CapEx and OpEx is necessary, which refer to the values expressed per mass unit (processing flow), which can be obtained assuming 8000 h as the annual operating time. In this sense, for the case evaluated, the specific CapEx is 0.12 kUSD/ton for both raw materials, and the specific OpEx is 0.82 and 0.54 kUSD/ton. Rueda et al. (2022) [19] reported the CapEx and OpEx of the pretreatment stage with H_2_SO_4_ using sugarcane bagasse as raw material. The specific CapEx and OpEx were 6.36 kUSD/ton and 1.53 kUSD/ton, respectively. The differences are due to the nature of the pretreatment in both cases. Sulfuric acid is much cheaper than TFA, even though the latter can be recovered. It causes an increase in CapEx for equipment (dimensions, as well as more equipment for recirculation) and in OpEx (cost of acids and quantities). Therefore, evaluating multiple economic pretreatments is necessary to establish a more complete and decisive analysis of TFA pretreatment performance [49]. However, economic analyses to complete processes such as fermentation to ethanol have been widely reported in the literature, but only a few have determined those analyses to yield C5 and C6 sugars. For example, Kumar and Murthy (2011) [50] reported a CapEx of 1.92 USD/L ethanol, using dilute acid as pretreatment to grass straw. An interesting comparison was found reported by Baral and Shah (2017) [9], in which the techno-economic comparisons of different pretreatments of corn stover are performed. The CapEx and OpEx were determined as a function of pretreatments of up to 113.5 ML/year of butanol. The results achieved with a steam explosion, sulfuric acid, AFEX, and biological pretreatment were 137, 125, 107, and 1508 MUSD for CapEx, 153, 145, 240, and 547 MUSD/year for OpEx, respectively. Compared to this paper, the cost of the pretreatment agent and the equipment involved in the pretreatment considerably influence the identification of the best economic alternative. However, it is necessary to continue evaluating and analyzing innovative processes, ideally from the technical, economic, and even environmental aspects.

TFA pretreatment of lignocellulosic materials has yet to be economically assessed. However, Baral et al. (2017) [9] reported cost distribution for different pretreatments. For example, for an acid pretreatment or steam explosion of corn stover, utilities represent more than 50%, mainly because of the energy demand. Additionally, they report that the detoxification stage represents 1% of the fermentable sugars production cost. However, the detoxification stage is unnecessary for TFA pretreatment, and the cost can be reduced. The results obtained for this paper agree with those reported in the literature, where it has been shown that utilities and depreciation contribute the most to the process costs [43,51]. Additionally, TFA pretreatment is economically competitive and comparable to other pretreatments. However, it is necessary to evaluate possible improvements and optimization to be applied in contexts where other pretreatments are already standardized. In this sense, joining efforts towards increasing the Technology Readiness Level (TRL) is striking and could complement this promising new technology until economic pre-feasibility is achieved.

## 4. Future Work

For future work, it is proposed to optimize the TFA pretreatment of lignocellulosic biomass, since it has not yet been attempted to evaluate several conditions of this process in the raw materials. In addition, it would be interesting to determine the environmental assessment of the schemes and understand the how the TFA pretreatment can affect different environmental indicators.

## 5. Conclusions

The TFA pretreatment of lignocellulosic biomass is a promising scheme to obtain a high yield of fermentable sugars (xylose and glucose). Compared to other pretreatments, the processing scheme does not require additional detoxification stages that could technically and economically affect the process, since it is an easily recovered acid. In energy terms, this pretreatment requires considerable thermal energy due to the temperatures of reaction and separation equipment. In this sense, it is proposed as an improvement to design energy integration processes within the scheme. Both raw materials evaluated showed a potential for valorization and the generation of value-added products through fermentable sugars and cellulose platforms. Both raw materials, are residues from agroindustrial and agroforestry processing. Thus, these feedstocks are interesting alternatives for obtaining C5 and C6 sugars, demanded by large biotechnological industries.

## Figures and Tables

**Table 1:** Equipment conditions and models used in the simulation with Aspen Plus V.9.0.

Equipment	Description	Conditions	Model
Dryer	Moisture removal of the raw material	1 bar, 60 °C	Dryer
Crusher-1	Primary mill, gyratory, particle size reduction to 2 cm	1 bar, 20 °C	Crusher
Screen-1	Separation of particles, size of screen opening 2 cm	1 bar, 20 °C	Screen
Crusher-2	Secondary mill, gyratory, particle size reduction to 1 cm	1 bar, 20 °C	Crusher
Screen-2	Separation of particles, size of screen opening 1 cm	1 bar, 20 °C	Screen
Mixer	TFA 2.5 M preparation	1 bar, 25 °C	Mixer
Pretreatment reactor	Reaction of hydrolysis to produce xylose and glucose mainly. Also considering the production of furfural and 5-HMF	2 bar, 120 °C	Heater and RStoic
Cooler	Reduction of the temperature to further purification the hydrolysate stream	1 bar, 30 °C	Heater
Centrifuge	Separation of solid and liquid fractions	1 bar, 30 °C	CFuge
Washer	Washing of the solid stream rich in cellulose and lignin fractions	1 bar, 26 °C	SWash
Evaporator	Moisture and acid remotion of the liquid fraction, rich in xylose and glucose	0.5 bar, 80 °C	Flash2

**Table 2:** Economic parameters used for the TFA pretreatment.

Item	Value	Conditions	Description
Operators wage	232.15	USD/month	Minimum wage 2022. Non-skilled person. RMR = 4812.37 COP = 1 USD (6 December 2022)
Supervisor wage	464.29	USD/month	High-skilled person. RMR = 4812.37 COP = 1 USD (6 December 2022)
Tax rate	33	%	[20]
Interest rate	13	%	[21]
CEPCI 2022	816.3	-	[22]
Operating time	350	days/year	Small-scale process
NCSB	0.015	USD/kg	[23]
PP	0.127	USD/kg	[24]
TFA	0.780	USD/kg	Cemotechnology-China (2022)
Processing water	0.326	USD/cum	[24]
LP steam	7.89	USD/ton	
MP steam	8.07	USD/ton	
Electricity	0.055	USD/kWh	
Cooling water	0.042	USD/cum	

**Table 3:** Proximate and elemental analysis of PP and NCSB.

Sample	Volatile Matter	Ash	Fixed Carbon	C %	H %	O %	Empirical Formula	Reference
PP	88.12	0.44	11.44	47.76	5.96	45.21	C_6_H_8.92_O_4.26_	This work
NCSB	85.96	1.05	12.99	47.69	5.91	44.71	C_6_H_8.87_O_4.22_	This work

**Table 4:** Sugar content on the liquid stream after TFA pretreatment, compared with other acid pretreatments.

Sample	Acid	Value	Arabinose	Galactose	Glucose	Xylose	Mannose	Reference
PP	TFA	Content (g/g raw material)	0.07 ± 0.01	0.16 ± 0.03	0.26 ± 0.04	0.26 ± 0.07	0.25 ± 0.06	This work
PR	H_2_SO_4_	Content (g/g raw material)	0.02	0.07	0.37	0.08	0.09	[33]
PT	H_2_SO_4_	Concentration (g/L)	2.96	7.90	16.93	4.5	29.82	[34]
NCSB	TFA	Content (g/g raw material)	0.17 ± 0.04	0.05 ± 0.01	0.31 ± 0.08	0.46 ± 0.06	0.01 ± 0.01	This work
SCB	H_2_SO_4_	Concentration (g/L)	1.14	N.R.	2.72	33.24	N.R.	[35]
SCB	H_2_SO_4_	Concentration (g/L)	7.90	6.44	4.38	25.57	9.64	[36]
SCB	HCl	Concentration (mg/L)	460	N.R.	20	40	N.R.	[37]
SCB	H_2_SO_4_	Content (mg/g raw material)	0.39	0.05	1.32	0.11	N.R.	[32]

PP: *Pinus patula* wood chips; PR: *Pinus Radiata*; PT: *Pinus Taeda*; NCSCB: Non-centrifuged sugarcane bagasse; SCB: Sugarcane bagasse; NR: Non-reported.

**Table 5:** Severity factor for pretreatments of some lignocellulosic raw materials.

Raw Material	Pretreatment	Acid Concentration	T (°C)	t (min)	Severity Factor	Reference
PP	TFA	2.5 M	120	120	2.67	This work
PP	Steam explosion	N.A	180	10	3.36	[38]
PR	HCl diluted	2 M	120	30	2.07	[42]
EU and EG	Diluted Sulfuric acid	4.5% *w*/*w*	175	15	3.05	[44]
NCSB	TFA	2.5 M	120	120	2.67	This work
SCB	Diluted sulfuric acid	8% *w*/*w*	90	400	2.31	[39]
SCB	Organosolv	N.A	220	120	5.61	[45]
SCB	Acid–ultrasound–thermal treatment	3% *v*/*v*	80	60	1.19	[46]
WS	TFA	10% *w*/*w*	60	960	1.80	[10]

PP: *Pinus patula* wood chips; PR: *Pinus radiata* wood chips; EU and EG: Hybrid of Eucalyptus (*Eucalyptus urophylla* × *Eucalyptus grandis*) wood chips; NCSCB: Non-centrifuged sugarcane bagasse; SCB: Sugarcane bagasse; WS: Wheat straw. N.A: Not applicable.

**Table 6:** Mass and energy indicators for TFA pretreatment of PP and NCSB.

Case	Mass Indicators				Energy Indicator
	$Y_{P}$ (kg product/kg raw material)	PMI (kg raw material/kg products)	MLI (kg waste/kg products)	RMI (% kg renewable feedstock/kg raw materials)	$S_{EC}$ (MJ/kg)
PP	0.22 (sugar-rich stream *)	11.71	10.71	13.23	207.67
	0.43 (cellulose–lignin stream **)				
NCSB	0.27 (sugar-rich stream)	11.71	10.70	13.92	190.93
	0.35 (cellulose–lignin stream)				

*: sugar-rich stream: C5 and C6 hydrolyzates sugars. **: cellulose–lignin stream: Solid stream rich in cellulose and lignin content.

**Figure A1:**
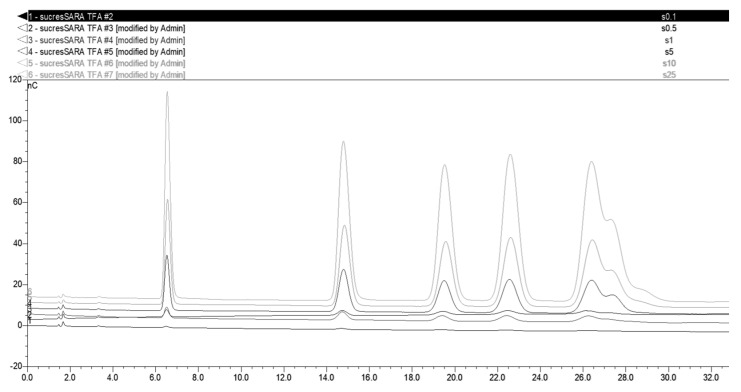
Chromatograms for sugar standards at different concentrations.

**Figure A2:**
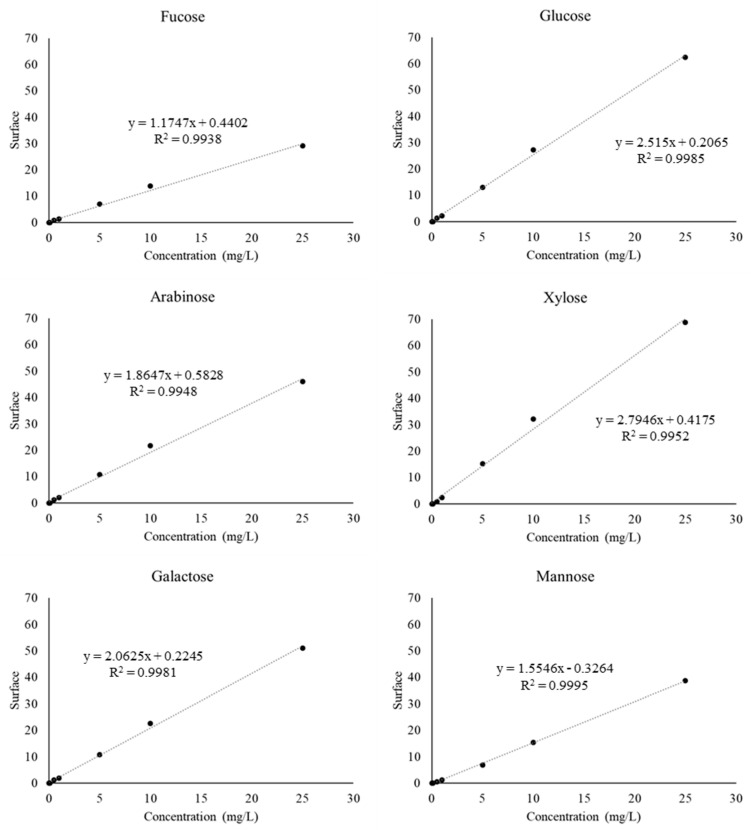
Calibration curves and linear regression equations for sugar standards.

## Data Availability

The data are available.

## Appendix A. Calibration curve and chromatograms for sugar standards

The sugar quantification included fucose, arabinose, galactose, glucose, xylose, and mannose. Figure A1 shows the chromatogram for each sugar quantification, at different concentrations (0.1, 0.5, 1.0, 5.0, 10.0, 25.0 g/L). Figure A2 shows the linear regression for each standard, and the related equation.
